# Motor Responses to Noxious Stimuli Shape Pain Perception in Chronic Pain Patients

**DOI:** 10.1523/ENEURO.0290-18.2018

**Published:** 2018-11-29

**Authors:** Henrik Heitmann, Elisabeth S. May, Laura Tiemann, Paul Schmidt, Moritz M. Nickel, Son Ta Dinh, Vanessa D. Hohn, Thomas R. Tölle, Markus Ploner

**Affiliations:** 1 Department of Neurology, Technische Universität München, Munich 81675, Germany; 2 TUM-Neuroimaging Center, Technische Universität München, Munich 81675, Germany

**Keywords:** behavior, chronic pain, motor, pain, perception

## Abstract

Pain serves vital protective functions, which crucially depend on appropriate motor responses to noxious stimuli. Such responses not only depend on but can themselves shape the perception of pain. In chronic pain, perception is often decoupled from noxious stimuli and motor responses are no longer protective, which suggests that the relationships between noxious stimuli, pain perception, and behavior might be changed. We here performed a simple experiment to quantitatively assess the relationships between noxious stimuli, perception and behavior in 22 chronic pain patients and 22 age-matched healthy human participants. Brief noxious and tactile stimuli were applied to the participants’ hands and participants performed speeded motor responses and provided perceptual ratings of the stimuli. Multi-level moderated mediation analyses assessed the relationships between stimulus intensity, perceptual ratings and reaction times for both stimulus types. The results revealed a significantly stronger involvement of motor responses in the translation of noxious stimuli into perception than in the translation of tactile stimuli into perception. This significant influence of motor responses on pain perception was found for both chronic pain patients and healthy participants. Thus, stimulus-perception-behavior relationships appear to be at least partially preserved in chronic pain patients and motor-related as well as behavioral interventions might harness these functional relationships to modulate pain perception.

## Significance Statement

Despite its frequent conceptualization as a perceptual phenomenon, the protective function of pain crucially depends on appropriate motor responses to potentially harmful stimuli. However, it is not fully clear how motor responses and pain perception relate to each other. The present study confirms that motor responses to noxious stimuli are significantly involved in shaping pain perception in healthy human participants. Moreover, the results reveal that similar effects can be observed in chronic pain patients. Thus, stimulus-perception-behavior relationships seem to be at least partially preserved in chronic pain patients. This can further the understanding of how behavioral therapies and motor-related stimulation techniques can be used to reshape the perception of pain in chronic pain patients.

## Introduction

Pain is commonly defined as “an unpleasant sensory and emotional experience associated with actual or potential tissue damage” (Merskey and Bogduk, 1994). Pain has, thus, been mostly conceptualized as a perceptual phenomenon. However, the crucial protective function of acute pain depends on appropriate behavioral responses rather than on perception. Accordingly, motivational and motor processes are increasingly recognized as important components of pain (Wall, 1979; Bolles and Fanselow, 1980; Fields, 2006; Sullivan, 2008; Morrison et al., 2013; Klein, 2015; Sullivan and Vowles, 2017; Tabor et al., 2017). However, it is not fully clear yet how behavioral responses and pain perception relate to each other.

Recently, a simple paradigm to quantitatively assess the relationships between noxious stimuli, pain perception, and behavioral responses has been established (May et al., 2017). In this paradigm, painful and non-painful stimuli are applied and relationships between intensity ratings as a measure of perception and reaction times as a measure of motor behavior in response to the applied stimuli are analyzed using moderated multi-level mediation analyses. More specifically, a more traditional view in which perception determines motor behavior (a perception-behavior model) and a more action-oriented view in which behavior determines perception (a behavior-perception model) are tested. Results from healthy participants revealed that motor responses to noxious stimuli not only result from, but also significantly shape the perception of pain.

In pathologic chronic pain states, perception is often decoupled from noxious stimuli (Baliki and Apkarian, 2015). Moreover, changes of psychological processes underlying pain-related behavior such as self-efficacy or coping strategies play an important role in chronic pain (Keefe et al., 2004; Gatchel et al., 2007; Crombez et al., 2012; Jackson et al., 2014; Edwards et al., 2016). These findings suggest that the relationships between noxious stimuli, pain perception, and behavioral responses are altered in chronic pain. However, quantifiable experimental evidence for an interaction between behavioral responses and pain perception in chronic pain is lacking so far.

Here, the previously established paradigm outlined above was used (May et al., 2017) to investigate whether stimulus-perception-behavior relationships are altered in chronic pain patients. The results show that motor responses significantly shape the perception of pain in both chronic pain patients and age-matched healthy participants. This evidence for at least partially preserved stimulus-perception-behavior relationships in chronic pain highlights that motor-related and behavioral interventions might not only change pain behavior but can also directly influence pain perception.

## Materials and Methods

### Participants

A total of 22 chronic pain patients (mean age ± SD: 60 ± 14 years, range 25–82 years, 19 females) and 22 age-matched healthy participants (60 ± 13 years, range 28–75 years, 18 females) participated in the experiment (Table 1). All participants were right-handed and gave written informed consent. The study was approved by the ethics committee of the Medical Faculty of the Technische Universität München and conducted in accordance with the relevant guidelines and regulations. Patients were recruited at the pain clinic of the university hospital of the Technische Universität München and via local support groups for chronic pain patients. Inclusion criteria comprised a clinical diagnosis of chronic pain, a duration of pain more than or equal to six months and a minimum reported average pain intensity ≥4/10 during the last four weeks (0 = no pain, 10 = worst imaginable pain). Patients with acute changes of the pain condition during the last three months, for example due to recent injuries or surgeries were excluded. All chronic pain conditions aside from predominant headache disorders were included (Table 1). Ten patients suffered from chronic widespread pain (CWP), nine patients predominantly suffered from chronic back pain (CBP) and/or joint pain (JP), and three patients from predominant neuropathic pain (NP). Mean duration of pain ± SD was 13 ± 9 years. A total of 17 patients reported regular intake of at least one pain medication (Table 1). Seven patients took selective serotonin or serotonin and noradrenalin reuptake inhibitors (SSRI/SSNRI), six patients non-steroidal anti-inflammatory drugs (NSAIDs) or opioids, four patients tricyclic antidepressants (TCA), and three patients GABAergic anticonvulsants. Medication was additionally quantified using the Medication Quantification Scale (MQS; see also Table 1; Harden et al., 2005).

**Table 1.** Clinical parameters of chronic pain patients

Patient	Age (y)	Gender	Pain duration (y)	Current pain intensity (VAS, 0-100)	Type of pain (predominant first if multiple)	Medication (MQS)	BDI	SF-MPQ sensory	SF-MPQ affective	STAI state	STAI trait	PD-Q	PDI
1	47	m	2	66	CBP	NSAID, opioid (10.3)	9	17	5	33	39	4	27
2	68	f	12	45	CBP	AH, GABAergic (7.8)	2	5	0	26	24	20	5
3	77	f	25	32	CWP	AH, NSAID (14.8)	7	24	9	31	21	23	3
4	65	f	16	73	CBP	SSNRI (7.8)	10	5	2	47	48	19	37
5	44	f	10	80	CWP	- (0)	17	17	5	35	35	31	44
6	39	f	12	18	CBP	TCA (2.3)	20	25	7	46	54	20	12
7	48	f	16	48	CWP	SSNRI, TCA (10.3)	9	19	5	32	36	8	37
8	67	f	2	72	NP	NSAID (3.4)	9	12	3	30	29	20	24
9	69	f	15	64	CWP	NSAID (3.4)	24	24	5	62	68	24	51
10	56	f	18	51	CWP	AH (8)	21	17	6	42	42	27	38
11	65	f	15	50	CWP	- (0)	11	11	6	37	45	20	24
12	72	f	10	81	CWP	AH, SSNRI (9.7)	9	24	6	36	49	17	33
13	69	f	20	54	CWP	AH, NSAID (15.1)	31	18	7	47	56	22	45
14	41	f	2	50	NP/CBP	GABAergic, opioid, SSNRI, TCA (32.9)	9	10	5	34	38	24	45
15	54	f	15	70	CWP	Opioid, SSNRI (9.1)	41	22	8	61	60	19	57
16	77	f	19	56	CWP	NSAID (5.6)	35	18	10	53	-	17	28
17	70	f	5	31	NP	Opioid, SSNRI (12.5)	10	11	2	32	35	17	9
18	82	f	3.5	0	CBP	Opioid, SSRI (8)	-	-	2	46	40	5	29
19	60	m	4	11	CBP	GABAergic, opioid (8.6)	20	3	2	45	47	12	28
20	57	m	23	48	JP/CBP	GABAergic, NSAID (7.2)	12	13	1	43	44	8	23
21	66	f	4	1	JP/CBP	AH (6)	2	4	0	27	24	7	5
22	24	f	8	70	CBP	TCA (4.6)	8	13	1	47	48	19	16
Mean	59.9		13	49			14.6	14.2	4.4	39.9	41.6	17.5	27.0
SD	14.4		9	24			10.5	7.5	2.9	10.4	12.0	7.1	15.2

f, female; m, male; CBP, chronic back pain; CWP, chronic widespread pain; JP, joint pain; NP, neuropathic pain; MQS, medication quantification scale; AH, antihypertensives; GABAergic, GABAergic anticonvulsant; NSAID, non-steroidal anti-inflammatory drug; SSRI, selective serotonin reuptake inhibitor; SSRNI, selective serotonin and noradrenalin reuptake inhibitor; TCA, tricyclic antidepressant; BDI, beck depression inventory II; PDI, pain disability index; PD-Q, painDETECT questionnaire; SF-MPQ, short-form McGill pain questionnaire; STAI, state-trait anxiety inventory; VAS, visual analog scale; -, none.

Healthy participants were recruited through advertisements on the university campus and at the university hospital. Inclusion criteria were right-handedness and capability to provide informed consent. Exclusion criteria comprised a past medical history of pain lasting for more than six months and any pain on the day of testing.

Exclusion criteria for both groups were sensory or motor deficits of the right upper extremity, e.g., caused by neuropathy or carpal tunnel syndrome, and neurologic and psychiatric diseases other than depression. Sensory and motor function was tested by a neurologist and unremarkable in all participants.

### Paradigm

**Figure 1.**
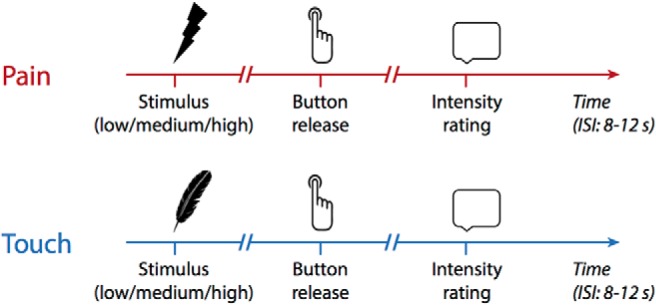
Paradigm. Pain and touch stimuli of varying intensity were applied to the right hand of healthy participants and chronic pain patients. Pain stimuli were brief cutaneous laser stimuli which selectively activate nociceptive afferents without activating tactile afferents. Touch stimuli were applied using von Frey-filaments steered by a computer-controlled device for standardized somatosensory stimulation. Presentation of pain and touch stimuli was pseudorandomly varied. Reaction times were measured as time from stimulus onset until the release of a button pressed with the stimulated hand. Perceptual ratings were obtained on numerical rating scales from 0 to 100. ISI, interstimulus interval.

To investigate the relationships between stimulus intensity, behavioral responses and perception in chronic pain patients and healthy participants, a previously established paradigm evaluating behavior by testing motor responses to noxious and non-noxious stimulation was used (Fig. 1; May et al., 2017). Brief painful heat and non-painful touch stimuli at three individually adjusted stimulus intensities were applied to the dorsum of the participants’ right hands while they were sitting in a comfortable chair with their eyes closed. The participant’s task was to react to each stimulus as fast as possible by releasing a button with the right index finger. Reaction times were taken as measures of behavioral responses to the stimuli. Subjects were further instructed to verbally identify the modality of the stimulus (pain or touch) and subsequently rate each stimulus on a numerical rating scale (NRS) ranging from 0 to 100. Rating scales were anchored at no pain and maximum tolerable pain for pain stimuli and at no touch and maximum non-painful touch for touch stimuli. Pain and touch ratings were taken as measures of perception. An equal importance of a fast reaction and a precise rating was emphasized during instructions.

Stimuli were applied in 4 blocks separated by short breaks. Within each block, stimulus modality (pain, touch) and stimulus intensity (low, medium, high; see below*)* were pseudorandomly varied with the constraints that no more than two stimuli of the same intensity and no more than three stimuli of the same modality were applied in a row. In each block, nine pain and touch stimuli at low, medium, and high intensities were applied. This resulted in 54 trials per block and a total of 108 pain and 108 touch trials for the whole experiment. Interstimulus intervals were randomly varied between 8 and 12 s. Preceding the first block, 18 practice trials were performed, subsequent blocks were preceded by six practice trials. Practice trials were not included in the analysis.

After completing the last block, *post hoc* ratings of the average stimulus intensity, unpleasantness and salience across all trials were obtained for pain and touch stimuli using visual analog scales (VAS 0-10) ranging from not intense/unpleasant/salient to highly intense/unpleasant/salient. Additionally, *post hoc* VAS ratings of task difficulty (very easy to very difficult, 0–10) and task preference (very focused on the reaction to very focused on the rating, –10 to 10) were obtained for touch and pain.

Stimulus presentation and timing was controlled using MATLAB (MathWorks) and the Psychophysics Toolbox (http://psychtoolbox.org/). Reaction times were recorded using a response box (MES Forschungssysteme GmbH), allowing an acquisition of response timing with millisecond accuracy.

### Stimuli

Pain stimuli were brief laser heat stimuli which selectively activate nociceptive afferents without concomitant activation of tactile fibers (Plaghki and Mouraux, 2005). Stimuli were administered using a Tm:YAG laser (Starmedtec GmbH) with a wavelength of 1960 nm, a pulse duration of 1 ms, and a spot diameter of 5 mm. A distance pin mounted to the hand piece of the laser device ensured a constant distance between skin surface and laser device. The stimulation site was slightly changed after each stimulus to avoid tissue damage.

Touch stimuli were applied using an in house-developed device employing von Frey-filaments to deliver phasic tactile stimuli to a small area of the skin (≤1 mm^2^) with a high precision of the applied intensity and timing (Dresel et al., 2008). Constant, logarithmically-scaled forces between 8 and 512 mN were used and stimulus duration was set to 80 ms.

Stimulation intensities of pain and touch stimuli were determined individually with the goal to elicit comparable ratings across subjects. The order of stimulus intensity determination (pain/touch or touch/pain) was counterbalanced across participants in both groups. Regression analyses were used to relate objective stimulus intensities to subjective ratings on the basis of 20 pain and touch stimuli of random intensities of each modality. Low, medium, and high stimulus intensities were selected, aiming at ratings of 30, 50, and 70 on the same NRS used during the experiments. Maximal stimulation intensities were 600 mJ and 512 mN for pain and touch stimuli, respectively. Resulting mean (±SD) stimulus intensities of low, medium, and high intensity pain stimuli were 479 ± 57, 525 ± 59, and 571 ± 62 mJ in patients and 490 ± 39, 535 ± 37, and 581 ± 36 mJ in healthy participants. For touch stimuli, mean stimulus intensities of low, medium, and high intensity stimuli were 188 ± 73, 290 ± 81, and 456 ± 91 mN in patients and 190 ± 68, 306 ± 60, and 505 ± 32 mN in healthy participants.

### Statistical analysis

Trials in which the stimulus modality (pain/touch) was not correctly identified or which yielded ratings of 0 were excluded from the analysis. Furthermore, trials with reaction times higher or lower than 2 SDs from the individual mean were excluded (Ratcliff, 1993). Resulting mean (±SD) trial numbers for pain stimuli of low, medium, and high intensity for patients/healthy participants were 29/32 (±1.0/0.6), 32/33 (±0.8/0.6), and 33/34 (±0.8/0.5), respectively. For touch stimuli of low, medium, and high intensity resulting mean (±SD) trial numbers for patients/healthy participants were 31/33 (±1.2/0.4), 33/33 (±0.8/0.3), and 33/34 (±0.8/0.3), respectively.

**Figure 2.**
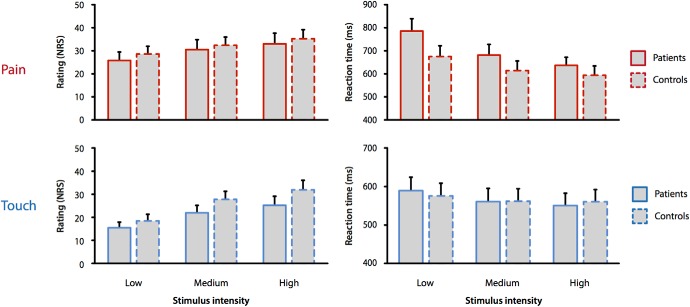
Perceptual ratings and reaction times to pain and touch stimuli in patients and controls. Mean ratings and reaction times for pain and touch stimuli of low, medium, and high intensities are shown. Error bars indicate the SE of individual means. Ratings increased and reaction times decreased with increasing intensity of pain and touch stimuli in patients and healthy control subjects.

To investigate whether stimulus intensity influenced perception and motor responses and to test for potential group- and modality-related differences in both measures, we first calculated repeated measures analyses of variance (ANOVAs) using SPSS (IBM SPSS, version 24.0). For both ratings and reactions times as dependent variables, repeated measures ANOVAs with the between subject factor group (patients vs controls) as well as the within subject factors stimulus intensity (low vs medium vs high) and modality (pain vs touch) were performed (Fig. 2).

**Figure 3.**
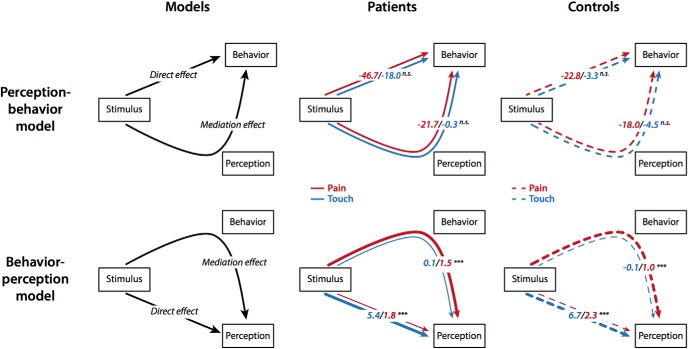
Moderated multi-level mediation analyses of the relationships between stimulus, behavior, and perception in patients and controls. Left, The perception-behavior-model reflecting the traditional view of stimulus-perception-behavior relationships (upper panel) and the behavior-perception-model reflecting an extension of the traditional view of relationships (lower panel). Middle, Results of the moderated multi-level mediation analyses for both models for pain and touch in chronic pain patients. In the perception-behavior-model (upper panel), the DE of stimulus on behavior and the ME of perception did not differ between modalities. In the behavior-perception-model (lower panel), the ME of behavior was significantly stronger for pain than for touch. Right, Results of the moderated multi-level mediation analyses for both models for pain and touch in healthy control subjects. Again, in the perception-behavior-model (upper panel), the DE of stimulus on behavior and the ME of perception did not differ between modalities. Like in chronic pain patients the behavior-perception-model (lower panel) shows that the ME of behavior was significantly stronger for pain than for touch. All effects are quantified in original units (milliseconds for behavior in the upper panel and ratings on the NRSs for perception in the lower panel) so that coefficients reflect the estimated average effects of a one level stimulation increase on the respective dependent variable. Effect sizes are further coded by the thickness of arrow lines. Significant differences between stimulus modalities (pain vs touch) are marked with asterisks. n.s., not significant; ***p < 0.001.

To further explore the relationships between stimulus intensity, motor responses and perception, we performed multi-level moderated mediation analyses (MacKinnon, 2013). Mediation analysis is a statistical approach, which quantifies the involvement of an intervening variable *M* called mediator in the effect of an independent variable *X* on a dependent variable *Y*. A variable *M* is a mediator, if *X* affects *Y* because *X* affects *M* and *M* affects *Y*. The total effect of *X* on *Y* in mediation analysis is composed of two effects [total effect = direct effect (DE) + mediation effect (ME); Fig. 3]. The DE represents the effects of *X* on *Y* independent of the mediator. The ME represents the effect of *X* on *Y* transmitted via the mediator. Moderated mediation analysis additionally quantifies how this ME changes in the light of an additional variable, the moderator.

Here, we performed mediation analyses using R (R Core Team, 2016; RRID:SCR_001905) and the lme4 (Bates et al., 2015; RRID:SCR_015654) and mediation (Tingley et al., 2014) packages. Analyses were performed on a x64-based PC using a Microsoft Windows 10 Pro operating system.

As in a previous study using the same paradigm (May et al., 2017), two different moderated mediation models were calculated (Fig. 3), which were tested for both the patient and the control group. Both models were composed of the predicting variable stimulus (operationalized by three levels of stimulus intensity) and the two response variables behavior (measured by reaction times) and perception (measured by ratings) which were included as either mediator or dependent variable. Importantly, the dependency of all effects on the moderating variable modality (pain or touch) was tested. The perception-behavior model (Fig. 3, upper panel) investigated the extent to which the effect of the stimulus on motor responses was mediated by perception in both modalities. Conversely, the behavior-perception model (Fig. 3, lower panel) investigated the extent to which the effect of the stimulus on perception was mediated by motor responses in both modalities. In both models, the magnitudes of DEs and MEs as well as the proportion of the ME relative to the total effect (proportion mediated = ME/total effect) were estimated and compared between the two levels of the moderator, i.e., between pain and touch stimuli. To investigate potential group differences, the proportion mediated was additionally compared between patients versus healthy controls for each model and modality.

In more detail, the following procedures were performed for both patients and healthy participants. Stimulus intensity was centered around 0. Reaction times and ratings were z-transformed across all trials and subjects for pain and touch stimuli separately to account for potential modality- or group-specific differences in reaction time and rating distributions. Subsequently, a set of linear mixed models was first fitted using the lmer function, quantifying the conditional distribution of the mediating variable *M* given the manipulation of the stimulus *X* and the conditional distribution of the outcome variable *Y* given the mediating variable *M* and the stimulus *X*. Random and fixed effects were modeled. Next, based on these models, estimates of DEs and MEs on both levels of the moderator modality were computed using the mediate function. Statistical inferences were based on the Monte Carlo method with 1000 simulations providing 95% confidence intervals of all estimates. Significance of the different effects (MEs, DEs, and proportion mediated) in each modality was inferred if the respective confidence interval did not include zero. Non-overlapping confidence intervals of the effects for pain and touch stimuli indicated a significant difference between modalities. For significantly different effects, exact *p* values were then obtained by subtracting the Monte Carlo samples for both modalities and examining the resulting distribution of differences (Tingley et al., 2014). For an easier grasp of the size of effects, all obtained coefficients were finally transformed back into original units to quantify the average effect of a one level stimulus intensity increase on reaction times and pain ratings (Fig. 3).

To test for statistical differences between patients and controls, the proportion mediated (ME/total effect) was compared between the two groups for both models (perception-behavior model/behavior-perception model) and modalities (pain/touch). For each of these four contrasts, the difference of the proportion mediated between the two groups (patients-controls) was calculated. Subsequently, patients and controls were randomly assigned to two groups, mediation analyses were repeated, and the difference of the proportion mediated was re-calculated. This was done 1000 times and resulted in a *p* value per model and modality, which was given by the proportion of permutations in which the difference of the proportion mediated exceeded the actually observed difference of the proportion mediated in the original groups.

*Post hoc* ratings of stimulus intensity, unpleasantness, salience, task difficulty and task preference were compared by performing repeated measures ANOVAs with the between subject factor group (patients vs healthy participants) and the within subject factor modality (pain vs touch).

Lastly, we controlled for a potential influence of medication intake on stimulation intensities, perceptual ratings and reaction times. To this end, we calculated Pearson correlations of individual patient MQS-scores quantifying pain-related medication on the one hand with averaged (across low, medium, and high intensity trials) stimulation intensities, perceptual ratings and reaction times for both modalities (pain and touch) on the other hand.

### Code accessibility and data sharing

The code described in the paper is freely available online at https://github.com/painlabmunich/Motor-responses-to-noxious-stimuli-shape-pain-perception-in-chronic-pain-patients. Raw data can be obtained from the corresponding author on request.

## Results

Figure 2 shows pain ratings and reaction times for the different stimulus intensities (low, medium, high), modalities (touch, pain), and groups (22 patients, 22 healthy participants).

To investigate whether stimulus intensity influenced perception and motor responses and to analyze potential group- and modality-related differences, repeated measures ANOVAs were performed. Testing the influence of stimulus intensity on reaction times showed significant main effects of intensity and modality (*F*_intensity(1,42)_ = 35.5; *F*_modality(1,42)_ = 15.1, both *p* < 0.001), but not of group (*F*_group(1,42)_ = 0.92, *p* > 0.05). In addition, significant two-way-interactions were found between modality and intensity (*F*_modality × intensity(1,42)_ = 17.7, *p* < 0.001) as well as group and intensity (*F*_group × intensity(1,42)_ = 5.7, *p* = 0.022). Inspecting the pattern of results, the effect of decreasing reaction times with increasing stimulus intensity was more pronounced for pain stimuli compared to touch stimuli and for patients compared to controls (Fig. 2, right panel). The corresponding analysis of perceptual ratings also revealed significant main effects of intensity and modality (*F*_intensity(1,42)_ = 49.2; *F*_modality(1,42)_ = 16.9, both *p* < 0.001), but not of group (*F*_group(1,42)_ = 0.78, *p* > 0.05), in combination with a significant two-way-interaction between modality and intensity (*F*_modality × intensity(1,42)_ = 10.7, *p* = 0.002). Thus, for both groups, the effect of increasing perceptual ratings with increasing stimulus intensity was more pronounced for touch stimuli compared to pain stimuli (Fig. 2, left panel). Overall, as expected, increasing stimulus intensities yielded faster behavioral responses and higher perceptual ratings for both modalities and groups. Before all following analyses, ratings and reaction times were z-transformed for each group and modality, which accounts for modality- and group-related differences in ratings and reaction times.

To further explore the relationships between stimulus intensity, motor responses and perception, we performed multi-level moderated mediation analyses (MacKinnon, 2013; Tingley et al., 2014). We first assessed whether perception mediated the effects of stimulus intensity on motor responses. We therefore calculated perception-behavior models for chronic pain patients and healthy participants (Fig. 3, upper panel). The results indicated that both MEs of perception and DEs of stimulus intensity on motor responses did not differ significantly between touch and pain. This was the case for both groups, i.e., chronic pain patients (ME_pain_: *β* = –0.07 [95% confidence interval: –0.11; –0.04]; ME_touch_: *β* = –0.002 [–0.05; 0.05]; DE_pain_: *β* = –0.15 [–0.23; –0.08]; DE_touch_: *β* = –0.11 [–0.19; –0.05]) and healthy participants (ME_pain_: *β* = –0.06 [–0.11; –0.03]; ME_touch_: *β* = –0.02 [–0.06; 0.01]; DE_pain_: *β* = –0.08 [–0.12; –0.04]; DE_touch_: *β* = –0.02 [–0.07; 0.03]). Taken together, in the perception-behavior model, perception mediated the effects of stimulus intensity on motor responses for touch and pain in both patients and healthy participants.

We further assessed whether motor responses mediated the effects of stimulus intensity on perception using the behavior-perception model (Fig. 3, lower panel). In this model, the ME of motor responses was significantly stronger for pain than for touch. This was similarly observed in patients (ME_pain_: *β* = 0.067 [0.033; 0.11]; ME_touch_: *β* = 0.007 [–0.004; 0.019]) and healthy participants (ME_pain_: *β* = 0.05 [0.02; 0.08]; ME_touch_: *β* = –0.005 [–0.014; 0.004]). In contrast, the DE of stimulus intensity on perception was stronger for touch than for pain in patients (DE_pain_: *β* = 0.08 [0.04; 0.12]; DE_touch_: *β* = 0.32 [0.18; 0.43]) and healthy participants (DE_pain_: *β* = 0.11 [0.07; 0.15]; DE_touch_: *β* = 0.34 [0.23; 0.45]). Consequently, the proportion of the ME of motor responses to the total effect of stimulus intensity on perception was significantly higher for pain than for touch. This was similarly observed in patients (pain, 46% [30.5; 63.1]; touch, 2% [–1.3; 6.0]) and healthy participants (pain, 29% [15.4; 41.9]; touch, –2% [–4.6; 1.0]).

To directly compare the MEs between patients and healthy participants, permutation-based statistics on the proportion mediated were performed. These analyses revealed no significant differences of the proportion mediated between the two groups for neither pain nor touch stimuli in the perception-behavior or behavior-perception model (all *p* > 0.05).

Additional correlation analyses of patients’ individual scores on the MQS with averaged stimulation intensities (*r*_pain_ = 0.12; *r*_touch_0.17; both *p* > 0.5), reaction times (*r*_pain_ = 0.018; *r*_touch_0.001; both *p* > 0.5) and perceptual ratings (*r*_pain_ = 0.16; r_touch_0.29; both *p* > 0.5) did not indicate an influence of medication intake on the analyzed parameters.

*Post hoc* ratings of intensity, unpleasantness, salience, task preference and task difficulty are shown in Table 2. ANOVAs showed that noxious stimuli were rated as more intense, more salient and more unpleasant than touch stimuli by both the patient and the control group. Task preference did not differ between groups or modalities. Task difficulty was rated higher by patients compared to healthy participants for both modalities.

**Table 2.** *Post hoc* ratings of stimulus and task characteristics for chronic pain patients and healthy controls

	Stimulus intensity		Stimulus unpleasantness		Stimulus salience		Task difficulty		Task preference	
	Pain	Touch	Pain	Touch	Pain	Touch	Pain	Touch	Pain	Touch
Patients mean (±SD)	5.5 (±2.4)	4.1 (±2.4)	4.7 (±2.8)	1.1 (±1.4)	7.2 (±1.8)	4.6 (±2.7)	3.7 (±2.7)	3.6 (±2.6)	-1.2 (±4.2)	-1.9 (±4.5)
Controls mean (±SD)	5.2 (±2.4)	3.6 (±2.8)	3.8 (±2.3)	0.8 (±1.1)	6.5 (±2.1)	4.7 (±2.4)	2.4 (±1.5)	2.4 (±2.1)	-0.3 (±2.6)	0.0 (±3.3)

For stimulus intensity/unpleasantness/salience, VAS was anchored at 0 = not intense/unpleasant/salient and 10 = highly intense/unpleasant/salient. For task difficulty, VAS was anchored at 0 = not difficult and 10 = very difficult. For task preference, VAS was anchored at –10 = very focused on the reaction and 10 = very focused on the rating. VAS, visual analog scale. For intensity, unpleasantness, and salience, ANOVAs showed a significant main effect of modality (*F*_intensity(1,40)_ = 18.7, *F*_unpleasantness(1,40)_ = 37.5, *F*_salience(1,40)_ = 24.9, all *p* < 0.001) but not of group (*F*_intensity(1,40)_ = 0.23, *F*_unpleasantness(1,40)_ = 0.55, *F*_salience(1,40)_ = 0.05, all *p* > 0.05) and no interactions between group and modality (*F*_intensity(1,40)_ = 0.15, *F*_unpleasantness(1,40)_ = 0.02, *F*_salience(1,40)_ = 0.06, all *p* > 0.05). For task preference, ANOVA did neither show a significant main effect of group (*F*_(1,42)_ = 1.3, *p* > 0.05) or modality (*F*_(1,42)_ = 1.1, *p* > 0.05) nor an interaction between group and modality (*F*_(1,42)_ = 2.4, *p* > 0.05). For task difficulty, ANOVA showed a significant main effect of group (*F*_(1,42)_ = 5.0, *p* = 0.031) but not of modality (*F*_(1,42)_ = 0.53, *p* > 0.05) and no interaction between group and modality (*F*_(1,42)_ = 0.42, *p* > 0.05).

Taken together, the present findings confirm that motor responses are significantly and pain-specifically involved in the translation of a noxious stimulus into the perception of pain. This effect of motor responses on perception was found in both chronic pain patients and healthy participants.

## Discussion

In the present study, we investigated stimulus-perception-behavior relationships in chronic pain patients and healthy participants. Using a previously established paradigm applying moderated mediation analyses to quantify the influence of perception on motor responses and vice versa (May et al., 2017), we found motor responses to shape the perception of noxious stimuli in both chronic pain patients and healthy participants. These findings suggest at least partially preserved stimulus-perception-behavior relationships in chronic pain patients. Moreover, such a partially preserved influence of behavior on perception might represent a mechanism by which motor-related and behavioral interventions can modulate pain perception in chronic pain patients.

The present results confirm previous findings in healthy participants, which found that motor responses are involved in the translation of noxious stimuli into perception (May et al., 2017). Together, these findings are in accordance with mounting evidence for more action-oriented concepts of pain emphasizing the importance of motor processes and behavior for pain processing (Wall, 1979; Bolles and Fanselow, 1980; Fields, 2006; Sullivan, 2008; Morrison et al., 2013; Klein, 2015; Sullivan and Vowles, 2017; Tabor et al., 2017). This acknowledgment of motor processes and behavior as important factors for pain perception parallels recent concepts of emotions, which assume that behavioral responses to threat shape emotional feelings (LeDoux, 2012; Damasio and Carvalho, 2013).

Moreover, we found that motor responses to noxious stimuli play a role in shaping pain perception in both chronic pain patients and healthy participants. This lack of a difference in stimulus-perception-behavior relationships between patients and healthy controls does not preclude that such a difference exists. Abnormal stimulus-perception-behavior relationships seem likely in the context of psychological factors such as helplessness, lack of self-efficacy and pain related fear-avoidance, which play an important role in the development and maintenance of chronic pain (Keefe et al., 2004; Gatchel et al., 2007; Crombez et al., 2012; Jackson et al., 2014; Edwards et al., 2016). However, these psychological factors as well as many structural and functional changes of the brain in chronic pain concern affective more than sensory processes (Baliki and Apkarian, 2015; Kuner and Flor, 2017). The paradigm of the present study in which behavioral responses were implemented as simple button releases can only partially reflect these complex relationships and the multifaceted nature of pain behavior, especially since it does not test an ecologically valid motor response such as withdrawal. However, in this context it appears likely, that button releases in response to noxious stimuli might be subserved by rather simple hard-wired sensorimotor pathways, which are not primarily affected by the adaptations taking place in pain chronification. Since we only obtained behavioral data, we can only speculate about the neuroanatomical and neurophysiological underpinnings of the observed effects. Motor areas of the cingulate cortex have been proposed as a pain-motor interface (Perini et al., 2013). As this area is a well-known source of laser-evoked potentials (Bradley et al., 2017), this is in accordance with findings relating defensive motor responses to noxious stimuli to vertex potentials (Moayedi et al., 2015). Future studies might further clarify the anatomic and physiologic substrates of pain-motor interactions in the brain. Moreover, they might address potential differences in the observed effects between body areas affected and unaffected by pain.

Taken together, our findings do not preclude changes of stimulus-perception-behavior relationships in chronic pain but indicate that some rather basic mechanisms underlying these relationships are preserved.

The present findings have potential implications for understanding pain therapy. First, preserved stimulus-perception-behavior relationships might provide a mechanism by which behavioral treatment interventions can modulate pain. Cognitive-behavioral interventions have been established in chronic pain treatment (Williams et al., 2012) and can reshape brain structure and function in chronic pain patients (Seminowicz et al., 2013; Shpaner et al., 2014). Moreover, interdisciplinary multimodal pain therapy approaches combining behavioral interventions on a physio- and psychotherapeutic basis such as exercise therapy and relaxation techniques are particularly effective in the treatment of chronic pain (Flor et al., 1992; Kaiser et al., 2017; Marin et al., 2017). Second, the influence of motor responses on pain perception might relate to the therapeutic effects of motor cortex stimulation on chronic pain, especially on NP (Lefaucheur et al., 2009; Nguyen et al., 2011; Lefaucheur, 2016) and CWP (Passard et al., 2007; Fagerlund et al., 2015). The underlying mechanisms are, however, only incompletely understood since motor cortex stimulation activates a large variety of adjacent as well as remote brain structures (DosSantos et al., 2016; Moisset et al., 2016). The present findings indicate that these behavioral and motor-related treatment approaches might not only influence pain behavior but that preserved stimulus-perception-behavior relationships might represent a mechanism by which they can directly modulate the perception of pain. Further studies are needed to elucidate the underlying neurophysiological mechanisms and to identify potential future therapeutic targets.

When interpreting the present results, the following limitations have to be taken into account. First, pain is inherently associated with a high salience and negative valence (Legrain et al., 2011). Based on the present results, it is therefore not possible to disentangle the effects of salience and valence from the effects of behavioral responses and their motivation, preparation and execution. Second, motor responses in the present study did not have a protective function as they did not prevent further stimuli or influence their intensity. Responses with a true protective function might yield different and possibly even stronger effects on perception. Third, in the present study motor responses were used as a proxy for behavior. However, a single parameter can only partially reflect such a complex and multifaceted construct. Fourth, due to a lack of power calculation approaches for the applied moderated multilevel mediation analyses, possible group differences at larger sample sizes cannot be ruled out.

In summary, the present results provide further evidence for a more action-oriented concept of pain perception and show that motor responses also shape the perception of pain in chronic pain patients. Together, these findings contribute to a better understanding of how motor-related and behavioral interventions might reshape the perception of pain in chronic pain patients.

Extended Data Figure 1. Software and code used for moderated mediation analyses. The code to perform moderated mediation analyses was generated with R, version 3.3.1 (2016-06-21). Additional R-packages installed were lme4 (Bates et al., 2015) and mediation (Tingley et al., 2014). The code was run on a x64-based PC using a Microsoft Windows 10 Pro operating system. Preprocessing of data was performed using MATLAB (MathWorks) to exclude trials where stimulus modality (pain/touch) was not correctly identified, trials which yielded ratings of 0 and trials with reaction times higher or lower than 2 SDs from the individual mean. Information on input data format is provided in the code as well as via a model file. Download Extended Data F, ZIP file.

## Synthesis

Reviewing Editor: Karen Davis, Krembil Research Institute and University of Toronto Decisions are customarily a result of the Reviewing Editor and the peer reviewers coming together and discussing their recommendations until a consensus is reached. When revisions are invited, a fact-based synthesis statement explaining their decision and outlining what is needed to prepare a revision will be listed below. The following reviewer(s) agreed to reveal their identity: NONE.

The study investigates the relationship between stimulus intensity, stimulus perception and motor response. The authors find no significant differences between patients and controls. While the findings are not entirely novel, the authors provide a compelling and quantifiable behavioural model to examine the relationship between stimulus, perception and behavioural outcomes in chronic pain patients. This manuscript provides a very basic framework to investigate how behavioural therapies and motor stimulation can mediate changes in pain perception in patients. Overall the study is straightforward, interesting, and well-designed, and the manuscript is well written and accessible

However the following issues should be addressed:

1. The study design is well balanced. It is obvious that the authors put a lot of effort in controlling all of the various parameters such as randomization of stimuli intensity and type. However, the following should be addressed regarding the statistical comparison performed:

a) A repeated measures ANOVAs was used with stimulus intensity as the independent variable and reaction times and pain ratings as dependent variables (Fig 2). They showed that increasing stimulus intensities resulted in faster behavioral responses and higher perceptual ratings for both modalities and groups. However to my knowledge the authors did not directly compare changes in perception and reaction time between groups or stimuli type. Was there a reason for not including stimulus type (noxious and touch) or subject type as categorical factors in the ANOVA test. If so the authors should justify their decision.

b) In the behavioral perceptual model the authors show that the mediation effect is significantly different between pain and touch. However there was no statistical analysis for patients vs healthy controls. Perhaps it is possible to perform some form of bootstrapping analysis. For example the authors could randomly assign patients and healthy subjects in 2 groups calculate the Direct effect (DE) and mediation effect (ME) and then calculate the differences between groups for the delta-DE)and delta-ME. Looping this for 5000 times will result in distributions of delta-DE and delta-ME that can be used to infer the p-value of delta-DE and delta-ME of the original patients and healthy groups. This is one example, perhaps the authors can use different methods to compare differences between healthy and patients.

2. In the intro and discussion, the authors need to better introduce and explain the two models and the significant differences between them. Although the authors provide a reference in the introduction, it remains unclear why do they use these models. Providing a better conceptual framework may allow the reader to understand the implications of the results.

3. In patients a lower stimulus intensity was given, and the ratings were slightly lower - could this have affected the outcome?

4. The statement of a central role for motor processes is not warranted - as it does not test an ecologically valid motor response (such as a withdrawal). Rather it tests a button release, and this is a goal-directed response to a stimulus or cue. Therefore, the authors can't make any claims about the central nature of motor responses to pain - rather they can discuss how pain may potentiate motor responses - but that's really it. This is acknowledged in the limitations, but should also be tempered elsewhere.

5. The authors should provide a power calculation based on the previous study to demonstrate that their sample size is adequate to identify (a) group effects and (b) group differences.

6. I'm very surprised the authors haven't cited previous very relevant work by the Iannetti group (Moayedi et al, Cerebral Cortex).

7. The authors focus on motor activity generated by M1 in the discussion. What about the cingulate motor area, which has been proposed as a pain-motor interface (see: Irene Strigo's work)

8. Could medication-use by patients have affected their sensory thresholds? How about the motor response? Was there a washout period?

9. Could there be a segment-specific effect for pain-motor interactions in patients? Would you expect the pain-motor relationship be altered should you stimulate or respond with an affected limb?

## Author Response

Response to referees

The study investigates the relationship between stimulus intensity, stimulus perception and motor response. The authors find no significant differences between patients and controls. While the findings are not entirely novel, the authors provide a compelling and quantifiable behavioural model to examine the relationship between stimulus, perception and behavioural outcomes in chronic pain patients. This manuscript provides a very basic framework to investigate how behavioural therapies and motor stimulation can mediate changes in pain perception in patients. Overall the study is straightforward, interesting, and well-designed, and the manuscript is well written and accessible

We thank the referees for carefully reviewing our manuscript and the positive assessment of the study.

However the following issues should be addressed:

1. The study design is well balanced. It is obvious that the authors put a lot of effort in controlling all of the various parameters such as randomization of stimuli intensity and type. However, the following should be addressed regarding the statistical comparison performed:

a) A repeated measures ANOVA was used with stimulus intensity as the independent variable and reaction times and pain ratings as dependent variables (Fig. 2). They showed that increasing stimulus intensities resulted in faster behavioral responses and higher perceptual ratings for both modalities and groups. However to my knowledge the authors did not directly compare changes in perception and reaction time between groups or stimuli type. Was there a reason for not including stimulus type (noxious and touch) or subject type as categorical factors in the ANOVA test. If so the authors should justify their decision.

We value this important comment and have now performed the suggested analyses. For both ratings and reaction times as dependent variables, repeated measures ANOVAs with the between subject factor group (patients vs. controls) as well as the within subject factors stimulus intensity (low vs. medium vs. high) and modality (pain vs. touch) were performed. Testing the influence of stimulus intensity on reaction times showed significant main effects of intensity and modality (Fintensity(1,42)=35.5; Fmodality(1,42)=15.1, both p&lt;0.001), but not of group (Fgroup(1,42)=0.92, p&gt;0.05). In addition, significant two-way-interactions were found between modality and intensity (Fmodality x intensity(1,42)=17.7; p&lt;0.001) as well as group and intensity (Fgroup x intensity(1,42)=5.7; p=0.022). Inspecting the pattern of results, the effect of decreasing reaction times with increasing stimulus intensity was more pronounced for pain stimuli compared to touch stimuli and for patients compared to controls (see Fig. 2 of the manuscript, right panel). The corresponding analysis of perceptual ratings also revealed significant main effects of intensity and modality (Fintensity(1,42)=49.2; Fmodality(1,42)=16.9, both p&lt;0.001), but not of group (Fgroup(1,42)=0.78, p&gt;0.05), in combination with a significant two-way-interaction between modality and intensity (Fmodality x intensity(1,42)=10.7; p=0.002). Thus, for both groups, the effect of increasing perceptual ratings with increasing stimulus intensity was more pronounced for touch stimuli compared to pain stimuli (see Fig. 2 of the manuscript, left panel). These analyses have now been added to the methods and results sections of the manuscript. Importantly, before subsequent mediation analyses, z-transformation of ratings and reaction times was performed separately for the two groups and modalities. Thereby, the main analyses of the present study accounted for potential modality- or group-specific differences in reaction time and rating distributions. This is now more strongly emphasized in the paragraph on statistical analysis of the methods section and in the results section of the manuscript.

b) In the behavioral perceptual model the authors show that the mediation effect is significantly different between pain and touch. However there was no statistical analysis for patients vs. healthy controls. Perhaps it is possible to perform some form of bootstrapping analysis. For example the authors could randomly assign patients and healthy subjects in 2 groups calculate the Direct effect (DE) and mediation effect (ME) and then calculate the differences between groups for the delta-DE and delta-ME. Looping this for 5000 times will result in distributions of delta-DE and delta-ME that can be used to infer the p-value of delta-DE and delta-ME of the original patients and healthy groups. This is one example, perhaps the authors can use different methods to compare differences between healthy and patients.

We thank the reviewers for this important suggestion. In line with the reviewers' comment, we have now calculated a statistical contrast of patients vs. healthy controls using a permutation-based approach. To capture each model in a single value, we contrasted the proportion mediated (mediation effect / total effect) between the two groups for each model (perception-behavior model / behavior-perception model) and modality (pain / touch). For each contrast, the difference of the proportion mediated of patients and healthy controls was calculated. Subsequently, patients and healthy controls were randomly assigned to two groups, mediation analyses were repeated, and the difference of the proportion mediated was re-calculated. This was done 1000 times and resulted in a p-value for each model and modality, which was given by the proportion of permutations in which the difference of the proportion mediated exceeded the actually observed difference of the proportion mediated in the original groups. This analysis did not reveal any significant differences between the two groups (all p&gt;0.05) and has now been included in the statistical analysis section of the methods as well as the results of the revised manuscript.

2. In the intro and discussion, the authors need to better introduce and explain the two models and the significant differences between them. Although the authors provide a reference in the introduction, it remains unclear why do they use these models. Providing a better conceptual framework may allow the reader to understand the implications of the results.

We appreciate this important comment and have added additional information for further clarification of the underlying concept and models in the introduction and discussion section of the manuscript.

3. In patients a lower stimulus intensity was given, and the ratings were slightly lower - could this have affected the outcome?

Stimulation intensities were individually adapted prior to experiments with the goal to elicit comparable individual ratings of 30, 50 and 70 for low, medium and high stimulus intensities, respectively. For comparisons of stimulation intensities between patients and controls, we have now performed additional ANOVAs with the between subject factors group (patients vs. controls) and the within subject factor stimulus intensity (low vs. medium vs. high) for both modalities. No significant main effects of group were found (Ftouch(1,42)=1.4 ; Fpain(1,42)=0.5; both p&gt;0,5). However, for touch stimuli only, a significant two-way-interaction between group and intensity was observed (touch: Fgroup x intensity(1,42)=5.1, p=0.03; pain: (Fgroup x intensity(1,42)=0.04, p=0.85) with higher stimulation intensities for high intensity touch stimuli for healthy controls than patients. Importantly, however, there were no group effects on perceptual ratings (please see our response to the first comment for the corresponding analysis). Thus, individually adapted stimulation intensities indeed differed slightly between groups regarding touch stimuli but, as intended, yielded comparable perceptual ratings. We therefore do not expect an influence on the outcome. The goal of individual adaptation of stimulus intensities is now stated more explicitly in the stimuli section of the methods.

4. The statement of a central role for motor processes is not warranted - as it does not test an ecologically valid motor response (such as a withdrawal). Rather it tests a button release, and this is a goal-directed response to a stimulus or cue. Therefore, the authors can't make any claims about the central nature of motor responses to pain - rather they can discuss how pain may potentiate motor responses - but that's really it. This is acknowledged in the limitations, but should also be tempered elsewhere.

We understand this concern and, in addition to explicitly addressing this limitation in the discussion section, have now tempered the claims about the central role of motor responses to pain. Moreover, we now more strongly emphasize that the button release in the present paradigm does not test an ecologically valid withdrawal response.

5. The authors should provide a power calculation based on the previous study to demonstrate that their sample size is adequate to identify (a) group effects and (b) group differences.

We appreciate this valuable comment and have discussed with statisticians and checked the literature again for power calculation for group comparisons of moderated multi-level mediation analyses. However, no established approach for such power calculations exist so that the power of our statistical comparisons remains undetermined. We thus cannot rule out that differences might be revealed at larger sample sizes. A corresponding paragraph has been added to the limitations section of the manuscript.

6. I'm very surprised the authors haven't cited previous very relevant work by the Iannetti group (Moayedi et al, Cerebral Cortex).

We have added a paragraph which discusses the study by Moayedi et al. and the possible neuroanatomical and neurophysiological underpinnings of the present findings to the discussion section of the manuscript.

7. The authors focus on motor activity generated by M1 in the discussion. What about the cingulate motor area, which has been proposed as a pain-motor interface (see: Irene Strigo's work)

We agree that the cingulate motor area might represent an important pain-motor interface and have added this point to the discussion section citing work by Irene Perini.

8. Could medication-use by patients have affected their sensory thresholds? How about the motor response? Was there a washout period?

Patients continued their regular pain medication and there was no washout period. Since stimulation intensities were individually adapted, we here use them as a proxy for sensory thresholds. To systematically test for potential influences of medication on all analyzed parameters, we have now calculated Pearson correlations between medication intake as quantified by the Medication Quantification Scale (MQS) (Harden et al., 2005) on the one hand and stimulus intensities, reaction times and ratings on the other hand. For neither pain nor touch stimuli, MQS-scores significantly correlated with averaged (across trials with low, medium and high) stimulation intensities (rpain=0.12; rtouch0.17; both p&gt;0.5), reaction times (rpain=0.018; rtouch0.001; both p&gt;0.5) or perceptual ratings (rpain=0.16; rtouch0.29; both p&gt;0.5). A significant influence of medication on sensory thresholds and motor responses is therefore unlikely but cannot ultimately be ruled out. These results have been added to methods and results section of the revised manuscript and individual MQS scores have been added to Table 1.

9. Could there be a segment-specific effect for pain-motor interactions in patients? Would you expect the pain-motor relationship be altered should you stimulate or respond with an affected limb?

Sensory and motor function of the right arm, which was used for both stimulation and response, was assessed by a clinical neurological exam prior to testing and unremarkable in all patients. It seems likely that, if an affected limb would be tested, alterations in the afferent (sensory) and/or efferent (motor) volley of the response assessed in the present paradigm would influence perception and/or reaction times. How this might then affect higher-order stimulus-perception-behavior relationships as addressed in our mediation analyses is an interesting question, which we cannot answer based on the present results. However, future studies comparing effects for affected and unaffected limbs are needed to address this aspect, which is now explicitly mentioned in the discussion.

References:

Harden RN, Weinland SR, Remble TA, Houle TT, Colio S, Steedman S, Kee WG (2005) Medication Quantification Scale Version III: update in medication classes and revised detriment weights by survey of American Pain Society Physicians. J Pain 6:364-371.
